# Discovering biomarkers for chronic sinusitis with nasal polyps: a study integrating bioinformatics analysis and experimental validation of macrophage polarization and metabolism-related genes

**DOI:** 10.3389/fbinf.2025.1613136

**Published:** 2025-09-15

**Authors:** Juan Zhou, Huan Wang, Jin Wang, Fuming Zhou

**Affiliations:** Department of Otolaryngology, Affliated Hospital of Yunnan University (Second People’s Hospital of Yunnan Province, Yunnan Eye Hospital), Kunming, Yunnan, China

**Keywords:** chronic rhinosinusitis with nasal polyps, macrophage polarisation, metabolism, machine learning, biomarkers

## Abstract

**Background:**

Macrophages play a critical role in chronic rhinosinusitis with nasal polyps (CRSwNP), and their functional imbalance may cause metabolic disturbances. However, the mechanisms of their role in CRSwNP remain unclear. This study aimed to identify CRSwNP biomarkers related to macrophage polarization and metabolism, and elucidate their molecular regulatory mechanisms.

**Methods:**

In this study, transcriptomic data of chronic rhinosinusitis with nasal polyps (CRSwNP) were obtained from public databases. Differentially expressed genes (DEGs) were screened via differential expression analysis. Subsequently, weighted gene co-expression network analysis (WGCNA) was used to identify key module genes related to macrophage polarization-related genes (MP-RGs), which were then cross-referenced with metabolism-related genes to screen for candidate genes. After that, two machine learning methods—least absolute shrinkage and selection operator (LASSO) and random forest (RF)—were applied to further screen these candidate genes. Receiver operating characteristic (ROC) curves for the training set and validation set were constructed, and gene expression validation was conducted to finally determine the biomarkers. Finally, reverse transcription-quantitative polymerase chain reaction (RT-qPCR) was used to verify the expression levels of prognostic genes.

**Results:**

ALOX5, HMOX1, and PLA2G7 were identified as biomarkers for CRSwNP, with AUC >0.7 in both training and validation sets, showing strong diagnostic potential. A nomogram, built on these three biomarkers, exhibited superior diagnostic performance. Enrichment analysis suggested that these biomarkers might be implicated in immune pathways. Furthermore, all three biomarkers were found to be correlated with asthma. Selenium was identified as a co-target of ALOX5 and HMOX1, presenting potential therapeutic targets for CRSwNP. A total of 10 key miRNAs regulating these biomarkers were identified, and the upstream long non-coding RNAs of hsa-miR-642a-5p, including FOXC1 and NEAT1, were predicted. Additionally, the transcription factor FOXC1 was found to concurrently regulate all three biomarkers. RT-qPCR results validated that the expression levels of ALOX5, HMOX1, and PLA2G7 were significantly elevated in CRSwNP patients, corroborating the findings from bioinformatics analyses.

**Conclusion:**

ALOX5, HMOX1, and PLA2G7 were identified as biomarkers linked to macrophage polarization and metabolism in CRSwNP. These findings offer new insights for early prevention strategies and clinical drug development in CRSwNP.

## 1 Introduction

Chronic rhinosinusitis (CRS) is a heterogeneous condition characterized by inflammation of the nasal cavity and paranasal sinuses, which is associated with a complex etiology and a high propensity for recurrence. Its prevalence is estimated at 10.9% among adults in European countries, 14% in the United States, and 8% in China (Chen M. et al., 2023). CRS imposes a significant economic burden on both patients and society. Nasal polyps are classified into two categories based on nasal endoscopy findings: chronic rhinosinusitis without nasal polyps (CRSsNP) and chronic rhinosinusitis with nasal polyps (CRSwNP) (Zhu et al., 2022). Defects in the sinus epithelial cell barrier, increased exposure to pathogenic and colonizing bacteria, and dysregulations in the host immune response have all been identified as key contributors to the pathogenesis of the disease (Chen G. et al., 2023). Given the incompletely understood pathogenesis of CRSwNP and its high recurrence rate, there is an urgent need to further elucidate its molecular and genetic mechanisms and expedite the development of targeted therapeutic approaches.

Macrophages are highly adaptable cells capable of sensing and responding to changes in their surrounding microenvironment. Upon activation, these cells are generally classified into two main types: classically activated (M1) macrophages and alternatively activated (M2) macrophages, based on their distinct functions. Both macrophages and monocytes, which fall under the category of phagocytic cells, play a pivotal role in both non-specific and specific immunity in vertebrates. M1 macrophages are activated by interferon-γ, lipopolysaccharide, and granulocyte macrophage colony-stimulating factor. They mediate Th1 immunity, produce pro-inflammatory mediators, and sustain inflammatory responses. Conversely, M2 macrophages are activated by IL-4, IL-13, IL-10, macrophage colony-stimulating factor, or transforming growth factor β. These macrophages elicit anti-inflammatory responses, promote tissue remodeling, and mediate Th2 immunity (Liu C. et al., 2022). Previous studies have demonstrated a significant increase in M2 macrophages within the nasal polyps of patients with CRSwNP (Zhu et al., 2022; Shaghayegh et al., 2022).

Metabolism is intricately linked to the maintenance of normal vital functions. Metabolic syndrome (MetS) encompasses a group of conditions that involve various metabolic abnormalities, including obesity, hyperglycemia, and dyslipidemia, among others. Research has indicated that MetS increases the risk of postoperative recurrence in patients with CRSwNP, with this risk rising proportionally to the number of MetS components. Additionally, studies have identified alterations in a range of metabolites, such as amino acids, fatty acids, and sugars, within the nasal mucosa of CRSwNP patients. These aberrant metabolic changes are implicated in the pathogenesis of CRSwNP, suggesting a potential connection between MetS, metabolite dysregulation, and the recurrence of this chronic condition (Chen Y. et al., 2024). Several studies have shown that macrophages are profoundly associated with metabolism. M1 and M2 macrophages represent two distinct extremes of macrophage activation. Imbalances between these two macrophage types can lead to a range of inflammatory and metabolic disorders (Qin et al., 2022; Wang T. et al., 2023). M1 macrophages exhibit a metabolic profile characterized by aerobic glycolysis, disruption of the tricarboxylic acid (TCA) cycle, and enhanced fatty acid synthesis. In contrast, M2 macrophages are believed to preferentially favor fatty acid oxidation and maintain a functional TCA cycle (He et al., 2020).

The preliminary research has revealed that few studies have explored the correlation between macrophage inflammatory response and metabolic pathways in CRSwNP. Therefore, this paper seeks to elucidate the prediction of macrophage alterations and to identify biomarkers related to metabolism in CRSwNP patients, as well as their molecular biological significance. By utilizing transcriptome data, the objective is to identify macrophage polarization-related genes (MP-RGs) and metabolism-related genes (MRGs), thereby providing new insights for the treatment of CRSwNP.

## 2 Materials and methods

### 2.1 Data source

Datasets GSE136825 and GSE194282, associated with CRSwNP, were retrieved from the Gene Expression Omnibus database (https://www.ncbi.nlm.nih.gov/gds). The GSE136825 dataset, which was accessed on 17 April 2024, based on the GPL20301 platform, included inferior turbinate tissue samples from 28 healthy controls and 75 CRSwNP patient samples, 42 of which were nasal polyp tissue samples and 33 were inferior turbinate tissue samples. This dataset served as the training set for the study. Ethical approval for this study was granted by the Ethics Committee of the Affiliated Hospital of Yunnan University (Second People’s Hospital of Yunnan Province, Yunnan Eye Hospital) (Approval number: 2024229), in compliance with the Declaration of Helsinki, and oral informed consent was obtained. The GSE194282 dataset, based on the GPL17692 platform, consisted of nasal polyp tissues from 7 CRSwNP patients and hook tissue samples from 7 healthy controls (accessed on 25 April 2024), and was utilized as the validation set (Zhou et al., 2023). Additionally, 35 MP-RGs and 2,752 metabolism-related genes (MRGs) were compiled from the literature (Zhao et al., 2021; Possemato et al., 2011) and were incorporated into this study.

### 2.2 Detection and identification of differentially expressed genes (DEGs)

The DESeq2 package (v 1.38.0) (Love et al., 2014) was employed to conduct differential expression analysis on CRSwNP and normal samples in the training set, with DEGs identified based on |log_2_Fold Change (FC)| > 1 and false discovery rate <0.05. Subsequently, the ggplot2 package (v 3.5.0) (Gustavsson et al., 2022) was used to generate volcano plots, and the pheatmap package (v 1.0.12) (Gu and Hübschmann, 2022) was utilized to create heat maps, highlighting the top 10 upregulated and downregulated genes sorted by log_2_FC.

### 2.3 Weighted gene co-expression network analysis (WGCNA) identified key modular genes associated with MP-RGs

The scores of MP-RGs in the training set were determined using the single-sample gene set enrichment analysis algorithm from the GSVA package (v 1.46.0) (Hänzelmann et al., 2013). Significant differences in these scores between CRSwNP and normal samples (*P* < 0.05) prompted the use of WGCNA through the WGCNA package (v 1.71) (Langfelder and Horvath, 2008) to analyze expression values associated with the MP-RGs scores. Hierarchical clustering was performed using Euclidean distances to detect potential outliers, which were subsequently removed. The MP-RGs scores were then re-clustered as sample traits. To construct the scale-free network, the best soft threshold was determined by ensuring that the scale-free fit index (*R* ^2^) was 0.9 and that the average connectivity was nearly zero. The minimum number of genes in each gene module was set to 100, according to the rules of the hybrid dynamic tree cutting algorithm. Gene similarity dendrograms were constructed to calculate the relationship between module feature genes and traits, and heatmaps were generated to visually depict these correlations. Finally, modules with a correlation to MP-RGs scores exceeding 0.4 and a *p*-value of less than 0.05 were selected as key modules. The genes within these key modules were identified as the key modular genes linked to MP-RGs.

### 2.4 Elucidation and functional characterization of candidate genes

Using the ggvenn package (v 0.1.9) (Zheng et al., 2022), the intersection of DEGs, MRGs, and key modular genes was identified. These intersected genes were subsequently utilized as candidate genes for further analysis.

Following the identification of the candidate genes, clusterProfiler software (v 4.7.1.003) (Yu et al., 2012) was used to perform Gene Ontology (GO) analysis and Kyoto Encyclopedia of Genes and Genomes (KEGG) pathway enrichment analysis. The GO analysis assessed biological processes (BP), cellular components, and molecular functions (MF). The top 10 enriched pathways were selected and displayed based on the results. To explore the interactions of the candidate genes at the protein level, the STRING database (http://string-db.org) (accessed on 17 April 2024) was utilized. This facilitated the construction of a protein-protein interaction (PPI) network, in which only interactions with a score above 0.4 were considered. The network was then visualized using Cytoscape software (v 3.8.2) (Liu et al., 2020). The Cytoscape plugin CytoNCA was employed to identify the most significant nodes in the PPI network. This process involved applying a range of parameters, including degree centrality, betweenness centrality, proximity centrality, eigenvector centrality, local average connectivity-based approach, and network centrality. The criterion established was that the threshold for each centrality metric was greater than the median. After applying these criteria, a PPI network containing the core genes was obtained.

### 2.5 Identification of signature genes through machine learning approaches

To identify genes closely associated with CRSwNP, a least absolute shrinkage and selection operator (LASSO) regression method penalized regression model was constructed using the glmnet package (v 4.1.4) (Li et al., 2022) based on the training set. With “CRSwNP disease status” as the dependent variable and the expression levels of core genes as independent variables, the regularization parameter λ was optimized through 10-fold cross-validation. Genes unrelated to CRSwNP had their coefficients shrunk (with coefficients approaching zero), while genes making significant contributions to classification were retained, thereby reducing the complexity of the model. Ultimately, genes with stable predictive ability were identified. Similarly, with “CRSwNP disease status” as the prediction phenotype, the randomForest package (v 4.7.1.1) (Alderden et al., 2018) was employed to carry out a random forest (RF) analysis. This analysis was evaluated using out-of-bag (OOB) error. When the number of decision trees reached approximately 600, the error stabilized, and the optimal number of trees was determined accordingly. The top 10 genes ranked by importance were selected for subsequent analyses. Additionally, to prevent overfitting, multiple decision trees were constructed by bootstrap sampling of samples and random selection of features, aiming to reduce the bias of individual trees. Finally, the ggvenn package was used to identify the signature genes for this study by analyzing the crossover results.

### 2.6 Identification of biomarkers and construction of a nomogram

To evaluate the ability of the signature genes to distinguish between CRSwNP and normal samples, the pROC package (v 1.74.0) (Robin et al., 2011) was employed to plot receiver operating characteristic (ROC) curves for these genes in both the training and validation sets. The area under the curve (AUC) for these ROC curves was subsequently calculated. Additionally, expression validation of signature genes was conducted in both the training and validation sets. Signature genes with an AUC >0.7, consistent and significantly different expression patterns across all datasets, were considered potential biomarkers. A nomogram was then constructed based on these biomarkers using the rms package (v 6.5.0) (Sachs, 2017). Each biomarker was assigned a specific score within the model, with the cumulative score of all biomarkers contributing to the total score. To assess the nomogram’s predictive performance, calibration curves were constructed. An ideal model would exhibit calibration curves with a slope approaching 1, indicating high accuracy in prediction.

### 2.7 Enrichment analysis of gene sets [gene set enrichment analysis (GSEA) approach]

To determine the biological pathways and functions of biomarkers in the training set, the psych package (v 2.2.9) (Author anonymous, 2023) was employed to evaluate the correlation between the biomarkers and other genes and to rank the correlation coefficients. KEGG gene annotations from the R package “org.Hs.eg.db” (v 3.16.0) (Qing et al., 2022) were used as the background, and the genes were matched to this set. Subsequently, GSEA was performed on the biomarkers using the clusterProfiler package to identify pathways that were significantly enriched (*P* < 0.05).

### 2.8 Construction of molecular regulatory networks

To explore the molecular regulatory mechanisms associated with the biomarkers, the miRDB (https://www.mirdb.org) and miRWalk (http://mirwalk.umm.uni-heidelberg.de/) databases (accessed on 17 April 2024) were utilized to predict miRNAs that interact with these biomarkers. The intersection of the predicted results from both databases was considered the set of key miRNAs. Based on the predicted key miRNAs, a miRNA-mRNA network was constructed. Subsequently, StarBase (http://starbase.sysu.edu.cn/index.php) (accessed on 17 April 2024) was used to predict the long non-coding RNAs (lncRNAs) that function upstream of these key miRNAs. An lncRNA-miRNA interaction network was then built to map the regulatory relationships. The two networks were subsequently integrated to form the lncRNA-miRNA-mRNA network. Additionally, to explore transcription factors (TFs) that target regulatory biomarkers, the JASPAR database (http://www.jaspar.genereg.net) (accessed on 17 April 2024) was used to identify TFs that regulate the biomarkers, and a TF-mRNA network was constructed. The results of these analyses were graphically represented using Cytoscape software.

### 2.9 Disease association and drug prediction analyses

To obtain biomarker-disease associations, biomarkers were imported into the Disease Gene Network database (http://www.disgenet.org/) (accessed on 17 April 2024) and analyzed, with a score >10 indicating an association. Additionally, to identify potential drugs targeting the biomarkers, relevant drug compounds were predicted using the Drug Gene Interaction Database (accessed on 17 April 2024). Subsequently, a drug-biomarker interaction network was delineated using Cytoscape software to visually represent the relationships between the drugs and biomarkers.

### 2.10 Validation of gene expression by reverse transcription-quantitative polymerase chain reaction (RT-qPCR)

The expression of these biomarkers was confirmed using RT-qPCR. Five pairs of CRSwNP and control tissue samples were obtained from the Affiliated Hospital of Yunnan University. Informed consent was obtained from all participants, and the study was approved by the hospital’s ethics committee (approval number: 2024229). Total RNA was extracted from 10 samples using TRIzol reagent (Ambion, Austin, United States) following the manufacturer’s guidelines. The quality and concentration of extracted RNA were verified using a NanoPhotometer N50, with OD260/280 ratios ranging from 1.8 to 2.0, indicating high RNA purity. Next, first-strand cDNA was synthesized from 1 μg of total RNA using the SweScript First-Strand cDNA Synthesis Kit (Servicebio, Wuhan, China) according to the manufacturer’s protocol: the reaction mixture (20 μL total volume) included 4 μL of 5× reaction buffer, 1 μL of reverse transcriptase, 1 μL of oligo (dT) primer, 1 μg of RNA template, and nuclease-free water to volume. RT-qPCR was performed using the 2 × Universal Blue SYBR Green qPCR Master Mix (Yeasen, China) on a CFX96 real - time PCR detection system (BIO - RAD, United States). The 10 μL reaction system contained 5 μL of 2xUniversal Blue SYBR Green qPCR Master Mix, 1 μL of forward primer (10 μM), 1 μL of reverse primer (10 μM), 3 μL of cDNA template. The thermal cycling conditions were: initial denaturation at 95 °C for 1 min, followed by 40 cycles of denaturation at 95 °C for 20 s, annealing at 55 °C for 20 s, and extension at 72 °C for 30 s. After the reaction was completed, specificity was verified via melting curve analysis. The specific primer sequences for target biomarkers and the internal reference gene are listed in Table 1. All primers were designed using NCBI Primer-BLAST. During design, the primers were ensured to span exon-intron boundaries to exclude genomic DNA contamination. Besides, glyceraldehyde-3-phosphate dehydrogenase (GAPDH) was used as the internal reference gene for data normalization. Finally, the relative expression levels of target biomarkers were calculated using the 2^(−ΔΔCt)^ method (Livak and Schmittgen, 2001), where ΔCt = Ct (target gene) - Ct (GAPDH), and ΔΔCt = ΔCt (CRSwNP group) - ΔCt (control group). Results are presented as fold changes relative to the control group.

**TABLE 1:** PCR primer sequence.

Primer	Sequence
ALOX5 F	CCAGACCATCACCCACCTTC
ALOX5 R	TGTCAAAGAGGCCACACTCG
HMOX1 F	GGGAATTCTCTTGGCTGGCT
HMOX1 R	GCTGCCACATTAGGGTGTCT
PLA2G7 F	GGGTGAATTCAGCAGGGAGT
PLA2G7 R	CTGGACCCGCGGTTAACTT
GAPDH F	ATGGGCAGCCGTTAGGAAAG
GAPDH R	AGGAAAAGCATCACCCGGAG

### 2.11 Statistical analysis

Statistical analyses for the bioinformatics component were performed using R software (version 4.2.2). For comparisons between two groups, the Wilcoxon rank-sum test was applied. For RT-qPCR data, relative expression levels were calculated using the 2^−ΔΔCt^ method and compared between groups using an unpaired t-testthe. In all statistical analyses, a two-tailed P-value <0.05 was considered statistically significant.

## 3 Results

### 3.1 Identification of 133 candidate genes in CRSwNP associated with macrophage polarisation and metabolism

**FIGURE 1:**
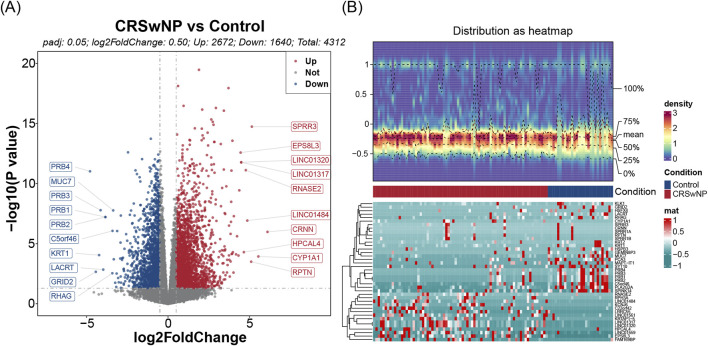
**(A)** Volcanograms of differential genes. The x-axis denotes log2 fold change (log2FC), and the y-axis denotes adjusted P-value. Blue dots represent downregulated genes, while red dots represent upregulated genes. The top 10 genes are labeled. **(B)** Heat maps of differential genes. The upper panel shows the expression density heatmap, the middle panel shows the grouping information, and the lower panel shows the gene expression heatmap.

**FIGURE 2:**
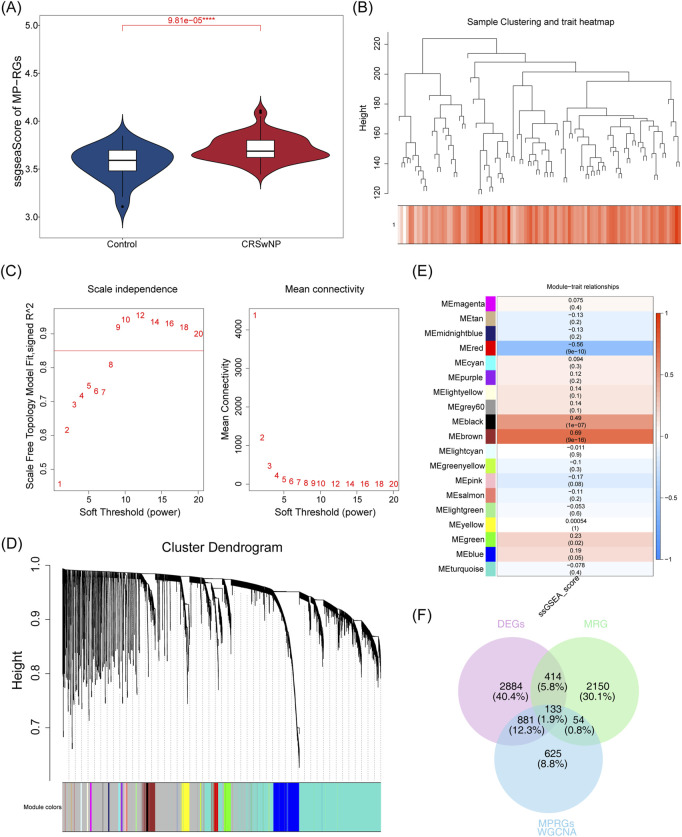
**(A)** Differences in MP-RGs gene set scores were analyzed between groups. Blue denotes the control group, and red denotes the CRSwNP group. **(B)** Sample cluster tree. Branches represent samples; the y-axis denotes the height of hierarchical clustering; the bottom of the figure shows the scoring status of samples. **(C)** Soft threshold filtering. Both x-axes denote the weight parameter power value; the y-axis of the left panel represents unscaled *R* ^2^, and the y-axis of the right panel represents the average connectivity of all genes in the corresponding gene modules. **(D)** Gene clustering dendrogram. The upper part shows the hierarchical clustering dendrogram of genes, and the lower part shows the gene modules. **(E)** Module - Correlation heat map of the disease. The x-axis denotes the scGSEA functional enrichment score, and the y-axis denotes the signature genes of each module. The values in parentheses below each module are, in order, the correlation coefficient (r) and statistical P-value. **(F)** Differential genes, MP-RGs direction genes, and MRG direction genes intersect venn diagram. Purple denotes DEGs, green denotes MRGs, and blue denotes MPRGs.

Differential expression analysis conducted on the training set identified 4,312 DEGs, of which 2,672 were upregulated and 1,640 downregulated (Figures 1A,B). Further assessment of the MP-RGs scores between the CRSwNP and normal samples revealed significantly higher MP-RGs scores in the CRSwNP samples compared to the normal controls (*P* < 0.0001) (Figure 2A). This finding suggests that macrophage polarization may play a critical role in the pathophysiology of CRSwNP. Consequently, WGCNA was employed to identify key modular genes associated with MP-RGs. As shown in the figure, no significant outliers were detected, and all samples were retained for subsequent analysis (Figure 2B). The optimal soft-threshold power was determined to be 9, where *R* ^2^ reached 0.9, and the average connectivity value approached zero (Figure 2C). A dendrogram was then constructed, leading to the identification of 19 modules (Figure 2D). The MEblack module (cor = 0.49), MEred module (cor = −0.56), and MEbrown module (cor = 0.69), which exhibited absolute correlation coefficients greater than 0.4 with the MP-RGs scores, were designated as key modules. These modules collectively comprised a total of 1,693 key modular genes (Figure 2E). Metabolic reprogramming was a key regulatory mechanism underlying macrophage polarization. Therefore, after screening 4,312 DEGs and 1,693 key modular genes, 2,752 MRGs were introduced for overlap analysis, with the aim of further focusing on candidate genes that were also involved in metabolic processes from the previously screened genes, we identified 133 candidate genes (Figure 2F).

### 3.2 Candidate genes related to functions in CRSwNP

**FIGURE 3:**
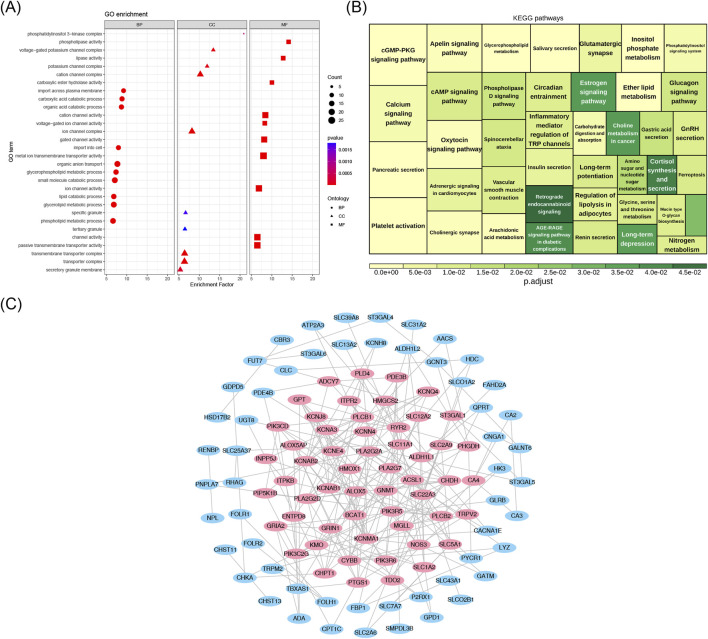
**(A)** Candidate gene GO analysis bubble map. The y-axis denotes pathway names, and the x-axis denotes the enrichment factor. Circles represent BP (Biological Process) terms, triangles represent CC (Cellular Component) terms, and squares represent MF (Molecular Function) terms. The size of the bubbles corresponds to the number of enriched genes. **(B)** KEGG pathway diagram. Color denotes significance; the legend indicates adjusted P-values; and the area size of the squares corresponds to the number of enriched genes. **(C)** PPI network. Nodes represent candidate genes, and edges indicate interactions; pink nodes represent core genes.

Among the 289 enriched GO-BP entries, significant associations were observed between candidate genes and functions such as “organic anion transport”, “small molecule catabolic process”, and “glycolipid metabolic process”. In the 28 enriched GO-CC entries, candidate genes were associated with structures such as the “ion channel complex”, “transmembrane transporter complex”, and “phosphatidylinositol 3-kinase complex”, among others. Within the 132 GO-MF categories, associations were found between the candidate genes and functions such as “metal ion transmembrane transporter activity”, “channel activity”, and “lipase activity”, among others (Figure 3A), indicating that the candidate genes might have acted by regulating the clearance of inflammatory mediators and the metabolic reprogramming of macrophages, thereby maintaining the chronic inflammatory microenvironment of the nasal mucosa; meanwhile, they might have synergistically participated in the signal transduction of immune cell activation and lipid metabolism disorders, promoting the abnormal proliferation of nasal polyp tissues and the persistence of inflammation. Additionally, these candidate genes were found to be enriched in 41 KEGG pathways, participating in various BPs, including metabolism, signaling, neurotransmission, and hormone secretion (Figure 3B), suggesting that candidate genes may act by participating in the crosstalk regulation of the nasal mucosal neuro-immune-endocrine-metabolic network, synergistically promoting the abnormal proliferation of nasal polyp tissues and the chronic persistence of inflammation. Furthermore, the PPI network analysis revealed interactions between the 133 candidate genes (Figure 3C). Subsequently, a total of 55 core genes were identified through screening with the CytoNCA plugin in Cytoscape (Figure 3C).

### 3.3 Machine learning identified five signature genes

**FIGURE 4:**
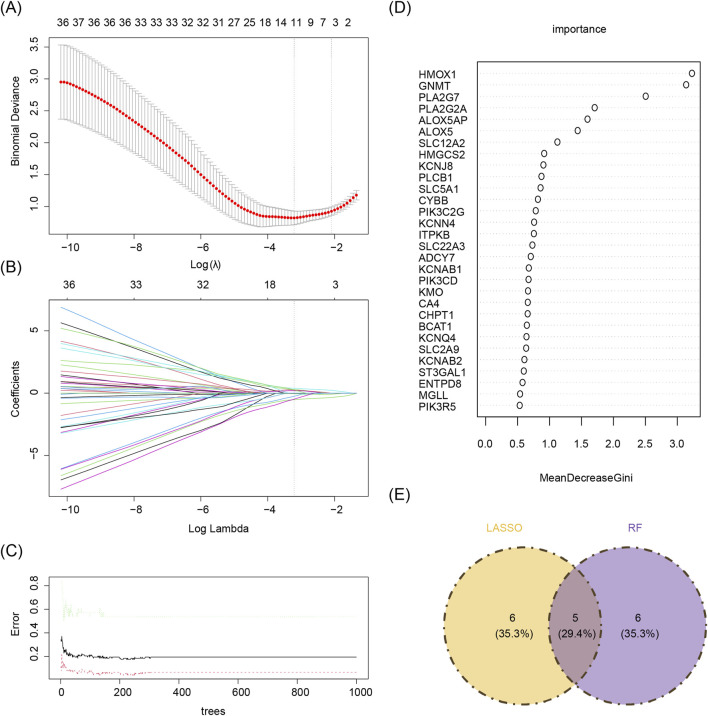
**(A,B)** Lasso regression analysis results. **(A)** 10-fold cross-validation for tuning parameters in LASSO analysis. The x-axis represents the logarithm of lambda, and the y-axis represents model error. The topmost x-axis indicates the number of variables. As the value of the parameter lambda increases, the number of independent variables decreases. **(B)** Lasso coefficient profile. The x-axis represents the logarithm of lambda, and the y-axis represents variable coefficients. Each line corresponds to a gene. **(C,D)** Random forest algorithm results for candidate gene screening. **(C)** The x-axis represents trees in the random forest, and the y-axis represents error rate. **(D)** The x-axis represents importance, and the y-axis represents genes. **(E)** Machine learning and random forests intersect. Yellow denotes the LASSO algorithm, and purple denotes the random forest algorithm.

Based on the 55 core genes, signature genes were further screened using LASSO regression and RF machine learning algorithms, respectively. A total of 11 genes, including PLA2G2A, NOS3, KCNAB1, ALOX5, PIK3C2G, HMOX1, CA4, PLA2G7, GNMT, BCAT1, and ITPKB, were selected from the 55 core genes by the LASSO regression algorithm, with lambda. min (0.09792358) as the optimal model (Figures 4A,B). The decision tree exhibiting the lowest error rate was derived using RF, and the importance of the genes was ranked. The top 10 genes identified were HMOX1, PLA2G7, SLC12A2, ADCY7, GNMT, PLA2G2A, ALOX5, KCNJ8, ALOX5AP, and MGLL (Figures 4C,D). Finally, the results from both algorithms were intersected to identify five signature genes: PLA2G2A, ALOX5, HMOX1, PLA2G7, and GNMT (Figure 4E).

### 3.4 Screening of biomarkers and construction a nomogram model for CRSwNP

**FIGURE 5:**
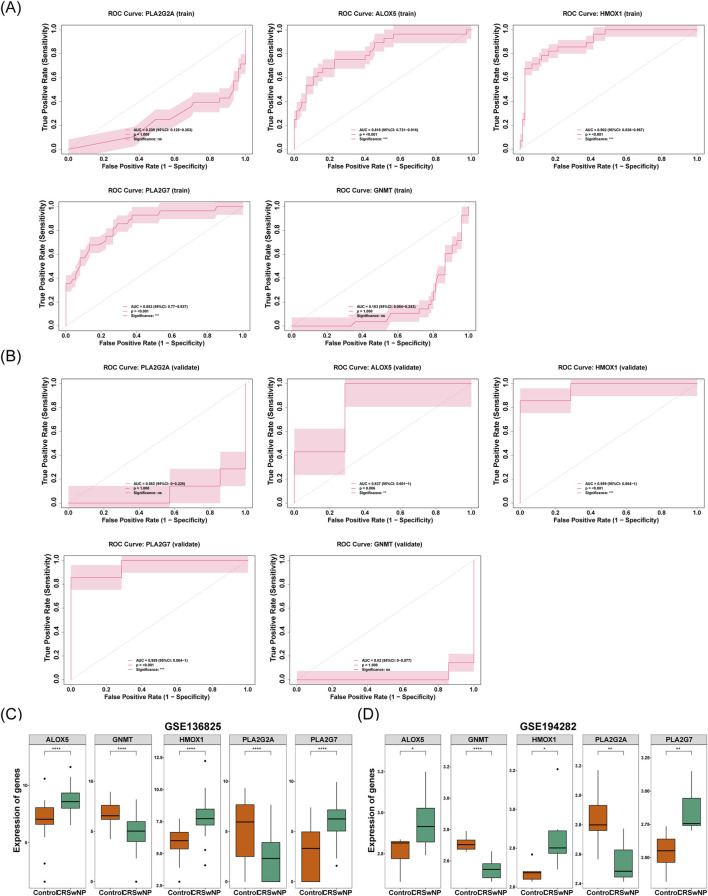
**(A)** Key genes in the ROC curve of the training set. The x-axis represents 1-specificity (false positive rate), and the y-axis represents sensitivity (true positive rate). Area Under the Curve (AUC), quantifying the predictive performance of genes for the outcome. From left to right, the genes are PLA2G2A, GNMT, PLA2G7, ALOX5, and HMOX1. **(B)** Key genes in the ROC curve of the validation set. The x-axis represents 1-specificity (false positive rate), and the y-axis represents sensitivity (true positive rate). Area Under the Curve (AUC), quantifying the predictive performance of genes for the outcome. From left to right, the genes are PLA2G2A, GNMT, PLA2G7, ALOX5, and HMOX1. **(C)** Differences in the expression levels of feature genes in the training set. The x-axis represents groups (orange: control group; green: CRSwNP), and the y-axis represents gene expression levels. **(D)** Differences in the expression levels of feature genes in the validation set. The x-axis represents groups (orange: control group; green: CRSwNP), and the y-axis represents gene expression levels.

The diagnostic value of the five signature genes was further analyzed and evaluated. The results demonstrated that ALOX5, HMOX1, and PLA2G7 exhibited AUC values of 0.818, 0.902, and 0.853, respectively. These values exceeded 0.7, indicating that these three genes possessed a strong capacity to differentiate between CRSwNP and normal samples. This is depicted in Figure 5A. Similarly, the AUCs of these three signature genes in the validation set surpassed 0.7, further corroborating their robust diagnostic potential (Figure 5B). To refine the identification of biomarkers, the expression levels of the five signature genes were assessed in both the training and validation sets. Notably, the expression of these three genes was significantly higher in the CRSwNP group compared to the normal group in both datasets (*P* < 0.05) (Figures 5C,D). This observation highlights the promise of these genes as diagnostic markers for CRSwNP, given their consistent and pronounced expression differences between the CRSwNP and normal groups across both datasets. Consequently, ALOX5, HMOX1, and PLA2G7 were identified as the biomarkers in this study.

**FIGURE 6:**
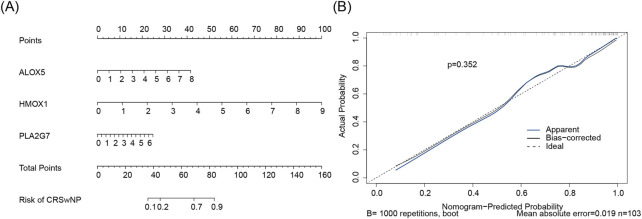
**(A)** Biomarker construction of nomogram. Points represent the score contributions corresponding to gene indicators. The scale adjacent to each gene denotes the measurement range of that gene, with vertical lines mapping these values to the respective Points above. Total Points reflect the model’s comprehensive quantification of risk. For the “Risk of CRSwNP” axis, the total score corresponds to the probability of CRSwNP via vertical lines. **(B)** Build a calibration curve for the line plot. The x-axis denotes the nomogram-predicted probability, and the y-axis denotes the actual probability. Ideal (dashed black line): Represents the perfect calibration state where predicted probability equals actual probability. Apparent (solid blue line): The uncorrected calibration curve, directly reflecting the model’s performance on the training set. Bias-corrected (solid black line): Calibration curve adjusted for overfitting via Bootstrap resampling, better approximating the model’s true performance on new data. Statistical indicators: p = 0.306 (Hosmer-Lemeshow test P-value, assessing calibration adequacy); Mean absolute error (MAE = 0.031, quantifying the average absolute difference between predicted and actual probabilities); n = 103 (sample size, reflecting data robustness).

Subsequently, a nomogram for CRSwNP was constructed based on ALOX5, HMOX1, and PLA2G7 (Figure 6A). The calibration curve of the nomogram exhibited a slope close to 1, demonstrating that the prediction accuracy of the nomogram was excellent (Figure 6B).

### 3.5 GSEA uncovers biomarker function and signaling pathways

**FIGURE 7:**
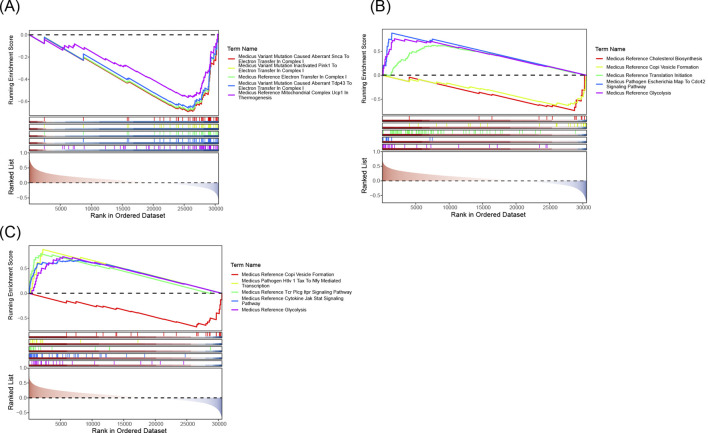
**(A)** ALOX5 GSEA analysis chart. **(B)** HMOX1 GSEA analysis chart. **(C)** PLA2G7 GSEA analysis chart. The five lines at the top are line graphs of gene Enrichment Scores. The y-axis corresponds to the Running Enrichment Score. The peak of each line graph represents the Enrichment Score of the gene set, and genes before the peak represent the core genes in this gene set. The x-axis represents each gene in this gene set.

GSEA analysis revealed that the biomarkers ALOX5, HMOX1, and PLA2G7 were significantly associated with 1,859, 2,228, and 2,025 pathways, respectively, in the training set. The top 10 pathways for each biomarker are presented in Figures 7A–C. Upon examining the significantly enriched pathways for all biomarkers (Supplementary Tables S1-S3), it was observed that they co-enriched into immune cell-related pathways, including ‘immunoregulatory interactions between a lymphoid and a non-lymphoid cell’ and ‘natural killer cell-mediated cytotoxicity’, among others. It was concluded that the three biomarkers may be strongly associated with inflammation and influence immunological pathways that impact the development and progression of CRSwNP.

### 3.6 Biomarkers are involved in complex molecular regulation

**FIGURE 8:**
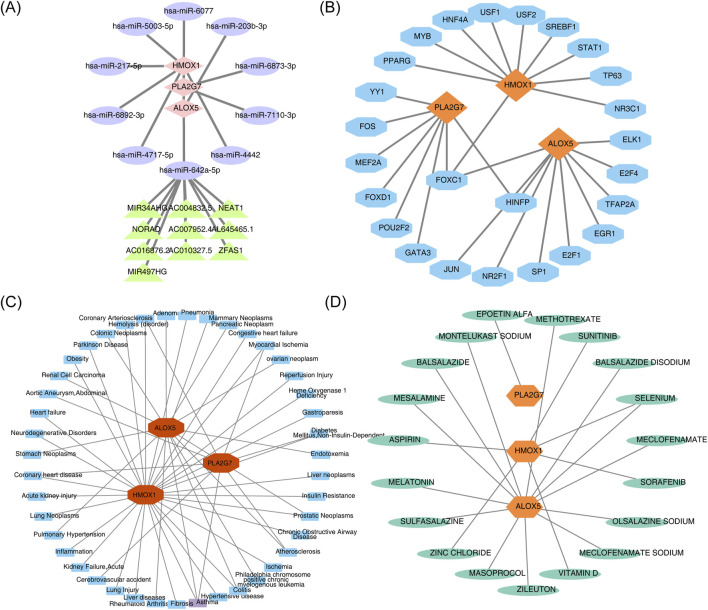
**(A)** LncRNA-miRNA-mRNA regulatory network. Pink diamonds represent biomarkers, purple ellipses represent miRNAs, and green triangles represent lncRNAs. **(B)** TF-mRNA (biomarkers) regulatory network. Orange represents biomarkers, and blue represents TFs. **(C)** Biomarker-disease network diagram. Blue denotes predicted diseases associated with the biomarker, while red denotes the biomarker. **(D)** Candidate gene targeted drug screening map. Green denotes predicted drugs associated with the biomarker, and orange-yellow denotes the biomarker.

Two databases were queried to predict key miRNAs potentially targeting the biomarkers, resulting in the identification of 10 key miRNAs corresponding to the three biomarkers. The miRNA-mRNA regulatory network revealed that no miRNAs targeted all biomarkers simultaneously (Supplementary Figure S1A). Subsequent predictions of the upstream lncRNAs for the 10 miRNAs identified that only hsa-miR-642a-5p predicted 10 lncRNAs, including FOXC1 and NORAD (Supplementary Figure S1B). A lncRNA-miRNA-mRNA regulatory network containing 22 nodes and 20 edges was further constructed, visualizing complex interactions such as ZFAS1-hsa-miR-642a-5p-PLA2G7 (Figure 8A). Additionally, the JASPAR database was utilized to identify TFs for the regulatory biomarkers, leading to the construction of a TF-mRNA regulatory network (Figure 8B). Among these, ALOX5 and HMOX1 were associated with the most TFs, and FOXC1 simultaneously regulated all three biomarkers.

### 3.7 Biomarkers could be used as targets for a variety of drug therapies

Analysis of the biomarker-disease network map revealed that ALOX5, HMOX1, and PLA2G7 were strongly associated with asthma (Figure 8C). Furthermore, a drug-biomarker interaction network was constructed by screening drugs from the DrugBank database. Examination of this network demonstrated that ‘selenium’ was identified as a co-target of both ALOX5 and HMOX1 (Figure 8D).

### 3.8 Expression verification of biomarkers

**FIGURE 9:**
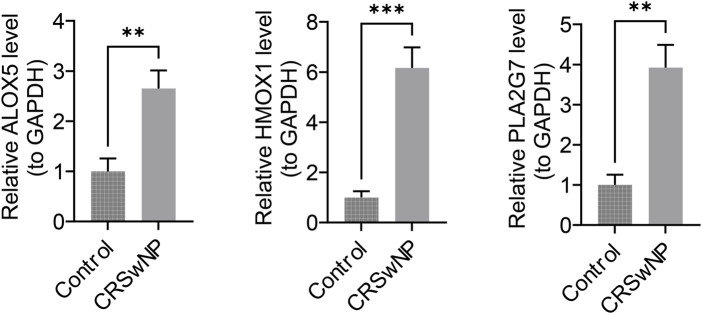
ALOX5, HMOX1, and PLA2G7 expression status. The x-axis denotes groups, and the y-axis denotes the relative expression levels of the biomarker.

The expression of biomarkers in clinical samples was further analyzed using RT-qPCR. Melting curve analysis revealed a single, sharp peak for ALOX5, HMOX1, PLA2G7 and the internal reference gene GAPDH, indicating specific amplification without non-specific products or primer-dimers (Supplementary Figure S2). The results demonstrated that the expression levels of ALOX5, HMOX1, and PLA2G7 were significantly higher in CRSwNP samples compared to control samples (*P* < 0.05) (Figure 9). These findings from the clinical sample validation are consistent with the results of the bioinformatics analysis, thereby enhancing the reliability of the bioinformatics approach.

## 4 Discussion

Research has demonstrated that in chronic sinusitis, an increase in M2 macrophage polarization contributes to the development of nasal polyps (Bao et al., 2023). Furthermore, metabolic disturbances influence the likelihood of postoperative recurrence in patients with CRSwNP (Chen Y. et al., 2024). Notably, a strong connection exists between macrophage polarization and metabolism (He et al., 2021), emphasizing the significance of investigating how these factors are related to CRSwNP.

In this study, candidate genes were selected by integrating MP-RGs, MRGs and CRSwNP DEGs, and three biomarkers (ALOX5, HMOX1, PLA2G7) were finally determined by machine learning, ROC analysis and expression level verification. Furthermore, PCR analysis confirmed their elevated expression levels in CRSwNP. Prior research has established a four-gene diagnostic model for CRSwNP, which includes HMOX1 and ALOX5 (Wang et al., 2024). Conversely, this study provides the first report linking PLA2G7 with CRSwNP.

Arachidonic acid lipoxygenase (ALOX) plays a critical role in catalyzing the conversion of polyunsaturated fatty acids to lipid hydroperoxides, a process that may lead to cell membrane damage and ferroptosis (Liu et al., 2023). Research on pancreatic cancer has demonstrated that ALOX5 can induce an M2-like phenotype in macrophages via the JAK/STAT pathway, thereby enhancing their chemotactic migration towards PANC-1 cells (Hu et al., 2023). In contrast, within the context of glioma, ALOX5 has been shown to facilitate disease progression by promoting 5-HETE-mediated immunosuppressive M2 polarization and elevating PD-L1 expression in glioma-associated microglia/macrophages (Chen T. et al., 2024). These studies collectively suggest that ALOX5-mediated macrophage M2 polarization, chemotactic enhancement, and immunomodulation are its core functions across diseases, and that nasal mucosal macrophages, as key regulators of the local inflammatory microenvironment of CRSwNP, may respond to ALOX5 regulation through similar mechanisms.

Heme oxygenase 1 (HMOX1) is an inducible enzyme that catalyzes the oxidation of cellular heme, resulting in the production of biliverdin, carbon monoxide, and free ferrous iron, a measurable indicator of oxidative stress (Schipper et al., 2009). Historically, HMOX1 has been acknowledged for its cytoprotective properties. However, growing evidence indicates that it can exhibit cytotoxic effects when its intracellular expression levels surpass a certain threshold (Zheng et al., 2023). In the context of CRSwNP, Wang et al. demonstrated that HMOX1 is highly expressed in M2 macrophages, with this expression positively correlating with eosinophil chemokine genes (Wang et al., 2024). Their study further revealed that the inhibition of HMOX1 expression results in a reduction in the number of M2 macrophages, suggesting a potential role for HMOX1 in macrophage polarization. It has also been confirmed that scRNA-seq data show that HMOX1 is localized to M2 macrophages, and HMOX1 may play a protective role in the pathogenesis of CRSwNP by regulating oxidative stress (Wang et al., 2025). Furthermore, HMOX1 has been implicated in various pathological processes, such as doxorubicin-induced ferroptosis in cardiomyopathy and the regulation of ferroptosis in diabetes-induced endothelial injury (Wu et al., 2022). Given its involvement in oxidative stress and inflammation, it is plausible that HMOX1 may also contribute to the metabolic disturbances observed in CRSwNP. The precise mechanisms by which HMOX1 modulates metabolism and macrophage polarization in CRSwNP remain to be fully elucidated. Therefore, further cell and animal studies are necessary to determine whether HMOX1 exerts cytoprotective or cytotoxic effects in this condition, as well as to explore its potential as a therapeutic target.

PLA2G7, also known as platelet-activating acetylhydrolase, belongs to the phospholipase A2 family and encodes the protein lipoprotein-associated phospholipase A2 (Lp-PLA2). This enzyme plays a pivotal role in catalyzing the hydrolysis of phospholipids, resulting in the release of free fatty acids and lysophospholipids (Huang et al., 2020). The significance of PLA2G7 is evident across a range of metabolic and inflammatory diseases, including atherosclerosis (Casas et al., 2010), diabetes (Canning et al., 2016), and autoimmune disorders (Li et al., 2021). In the context of CRSwNP, PLA2G7 may also assume a crucial role. Given its involvement in phospholipid metabolism and its association with inflammatory processes, it is plausible that PLA2G7 may influence the metabolic disturbances and macrophage polarization observed in this condition. Recent studies have highlighted PLA2G7’s potential as a promising biomarker in various diseases. In one study on hepatocellular carcinoma, it was shown that macrophages expressing PLA2G7 form a subset of highly immunosuppressive cells that hinder the activation of CD8 T cells (Zhang et al., 2024). Similarly, in CRSwNP, PLA2G7-expressing macrophages may contribute to the immunosuppressive microenvironment, thereby impeding effective immune responses. Therefore, further investigation into the role of PLA2G7 in CRSwNP is required to elucidate its potential impact on metabolism, macrophage polarization, and disease pathogenesis.

ALOX5, HMOX1, and PLA2G7 are genes that have been implicated in asthma. Research findings indicate that approximately 25% of patients with CRS also suffer from asthma, in contrast to only 5% of the general population. Notably, the association between asthma and CRS becomes more pronounced in patients with CRSwNP, where the prevalence of asthma increases to 30%–70%. This subgroup of CRS patients also experiences a more severe and progressive form of asthma (Galletti et al., 2023). Abdo et al. demonstrated that Anatabine can alleviate ovalbumin-induced asthma by reducing oxidative stress and inflammation, and by activating the Nrf2/HO-1 signaling pathway (Abdo et al., 2022). Similarly, Liu et al. found that 18β-glycyrrhetinic acid can suppress allergic airway inflammation in asthmatic mice by modulating the NF-κB and Nrf2/HO-1 signaling pathways (Liu J. et al., 2022). These findings highlight the potential dual role of HMOX1 in the airways, as it may both promote and attenuate inflammatory responses. Previous research has indicated that polymorphisms in the ALOX5 promoter can influence the response to asthma treatment (Mougey et al., 2013). However, despite the increasing interest in the role of these genes in asthma, there remains a lack of studies examining the relationship between PLA2G7 and asthma. Future investigations in this area could provide valuable insights into the pathogenesis and treatment of asthma.

GSEA analysis revealed that the three biomarkers were enriched in pathways associated with immune cell interactions, particularly in “immunomodulatory interactions between lymphocytes and non-lymphocytes” and “natural killer cell-mediated cytotoxicity”, among others. Previous studies have shown that the proportion of CD8^+^ T cells in the sinuses of patients with CRSwNP is elevated compared to the control group. However, no corresponding increase was observed in the percentage of NK cells. Additionally, both CD8^+^ T cells and NK cells in the sinus tissue displayed decreased levels of granzyme B and perforin. Based on these observations, it has been hypothesized that these three biomarkers may exert inhibitory effects on lymphocyte- and NK cell-mediated cytotoxicity. However, further investigation is needed to elucidate their specific mechanisms of action (Smith et al., 2017).

FOXC1 has been found to simultaneously regulate the three biomarkers. As a TF within the FOX family, FOXC1 is defined by a conserved “forkhead” or “wing helix” DNA-binding domain and is involved in various cellular processes, including growth, metabolism, and survival. Prior research has indicated that FOXC1 acts as a hypoxia-activated TF, thereby facilitating cancer cell proliferation. Moreover, several studies have reported that FOXC1 expression can be induced under conditions of myocardial ischemia (Chen et al., 2021). In a study involving cardiomyocytes from patients with heart failure, FOXC1 overexpression was shown to induce cellular senescence, as well as mitochondrial and systolic dysfunction (Li et al., 2024). The dual, potentially beneficial and detrimental, roles of FOXC1 in CRSwNP remain to be further elucidated.

The predictive analysis of drugs in this study showed that SELENIUM was predicted by both HMOX1 and ALOX5. As an essential trace element for the human body, selenium is the core cofactor of glutathione peroxidase (GPx), which can scavenge intracellular reactive oxygen species (ROS) and lipid peroxides, and reduce the damage caused by oxidative stress to tissues (Maia et al., 2024). It can also inhibit the release of pro-inflammatory factors such as IL-6, TNF-α, and IL-13 by regulating inflammatory signaling pathways such as NF-κB and MAPK, and regulate the activation and infiltration of immune cells such as eosinophils and T cells, and at the same time promote epithelial cell repair to enhance the integrity of the mucosal barrier and reduce the intrusion of external stimuli (Huang et al., 2012; Wang S. et al., 2023; Carlson et al., 2010; Zhao et al., 2024). The core pathological mechanism of CRSwNP involves oxidative stress imbalance, chronic hyperinflammation and epithelial barrier disruption (Yang et al., 2022; Toppila-Salmi et al., 2025), which clearly intersects with the above functions of selenium. Studies have confirmed that the combination of vitamin E and selenium can regulate allergic mediators and symptoms of rhinitis and asthma, reduce lung inflammation and airway mucus secretion, and help relieve bronchial obstruction (Jiang et al., 2021). Another study showed that normal saline combined with selenium-rich hot spring water could reduce lipopolysaccharide-induced inflammatory activity and hypersecretion of mucus in rats with sinusitis (Kim and Yeo, 2013), further supporting the potential role of selenium in related inflammatory diseases. In conclusion, it is speculated that selenium may be involved in the pathological process of -CRSwNP by targeting the oxidative stress-inflammatory pathway mediated by HMOX1 and ALOX5, providing new molecular targets and strategies for the prevention and treatment of CRSwNP.

## 5 Conclusion

In conclusion, this study investigated the correlation between CRSwNP and macrophage polarization and metabolism. Additionally, the expression of three biomarkers was validated, thereby establishing a foundational basis for subsequent experimental work. Nonetheless, there are still some shortcomings in this study. First, the small sample size in RT-qPCR validation may limit the generalizability of the results, and subsequent studies will expand the sample size to verify biomarker expression. Secondly, this study has not yet deeply elucidated the association between biomarkers and nasal mucosal macrophages, and more nasal mucosa-specific immunohistochemistry or functional experimental validation is still needed, and subsequent studies will focus on this direction.

## Data Availability

The datasets [GSE136825 and GSE194282] analysed during the current study are available in the Gene Expression Omnibus (GEO) database repository: https://www.ncbi.nlm.nih.gov/geo/query/acc.cgi?acc=GSE136825; https://www.ncbi.nlm.nih.gov/geo/query/acc.cgi?acc=GSE194282.

## Supplementary material

SUPPLEMENTARY FIGURE S1
**(A)** Predicted results of miRNA. **(B)** Predicted results of lncRNA. Pink diamonds represent biomarkers, purple ellipses represent miRNAs, and green triangles represent lncRNAs.

SUPPLEMENTARY FIGURE S2 Melting curve and Amplification curves. For the amplification curve, the x-axis denotes the number of cycles, and the y-axis denotes relative fluorescence units (RFU). For the melting curve, the x-axis denotes temperature ( °C), and the y-axis denotes the negative derivative of the fluorescence change rate.
