# Supplementary figures and images for: Discovering biomarkers for chronic sinusitis with nasal polyps: a study integrating bioinformatics analysis and experimental validation of macrophage polarization and metabolism-related genes
Source: Front Bioinform. 2025 Sep 15;5:1613136. doi: 10.3389/fbinf.2025.1613136 (PMC12477252; doi:10.3389/fbinf.2025.1613136)

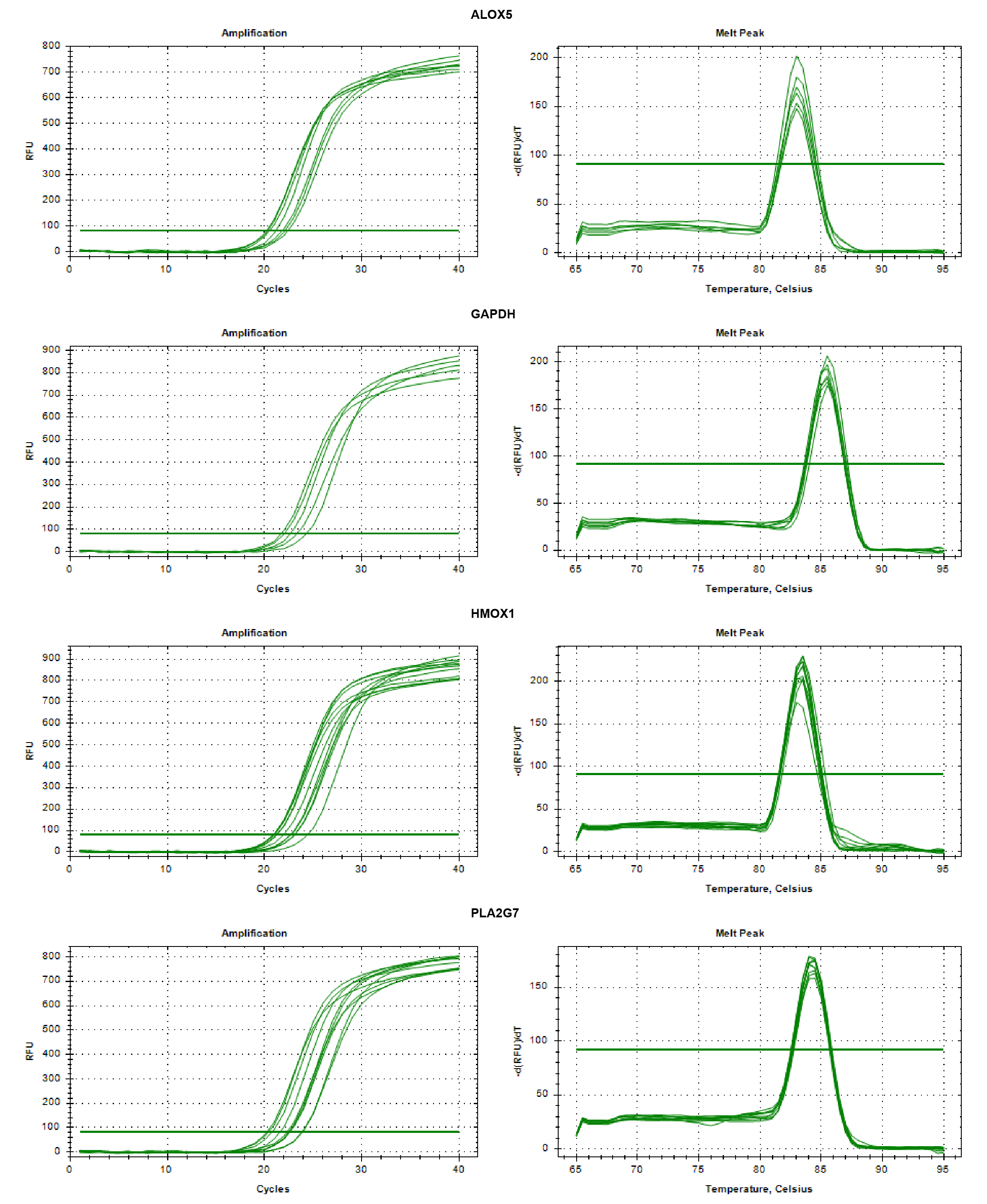

Supplement: Supplementary file 3 [file Image2.tif]

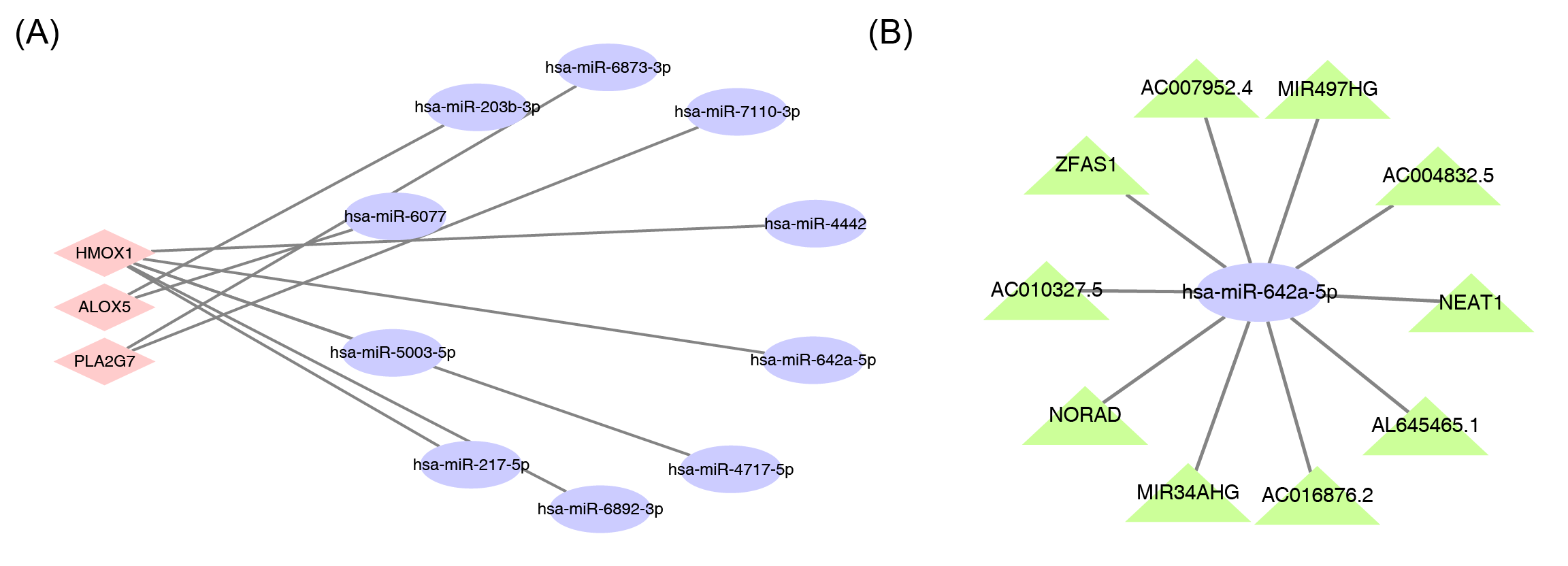

Supplement: Supplementary file 4 [file Image1.tif]
